# A radioenhancing nanoparticle mediated immunoradiation improves survival and generates long-term antitumor immune memory in an anti-PD1-resistant murine lung cancer model

**DOI:** 10.1186/s12951-021-01163-1

**Published:** 2021-12-11

**Authors:** Yun Hu, Sébastien Paris, Hampartsoum Barsoumian, Chike O. Abana, Kewen He, Duygu Sezen, Mark Wasley, Fatemeh Masrorpour, Dawei Chen, Liangpeng Yang, Joe D. Dunn, Saumil Gandhi, Quynh-Nhu Nguyen, Maria Angelica Cortez, James W. Welsh

**Affiliations:** 1 Department of Radiation Oncology, The University of Texas MD Anderson Cancer Center, 6565 MD Anderson Boulevard, Houston, TX 77030 USA; 2 Department of Translational Science, Nanobiotix, Paris, France; 3 Department of Radiation Oncology, Shandong Cancer Hospital and Institute, Shandong First Medical University and Shandong Academy of Medical Sciences, Jinan, China; 4 Department of Radiation Oncology, Koc University School of Medicine, Istanbul, Turkey

**Keywords:** Nanoparticle, Metastatic lung cancer, Radioimmunotherapy, Checkpoint blockade, Immune memory, NBTXR3, Radiation enhancer, Radiotherapy

## Abstract

**Background:**

Combining radiotherapy with PD1 blockade has had impressive antitumor effects in preclinical models of metastatic lung cancer, although anti-PD1 resistance remains problematic. Here, we report results from a triple-combination therapy in which NBTXR3, a clinically approved nanoparticle radioenhancer, is combined with high-dose radiation (HDXRT) to a primary tumor plus low-dose radiation (LDXRT) to a secondary tumor along with checkpoint blockade in a mouse model of anti-PD1-resistant metastatic lung cancer.

**Methods:**

Mice were inoculated with 344SQR cells in the right legs on day 0 (primary tumor) and the left legs on day 3 (secondary tumor). Immune checkpoint inhibitors (ICIs), including anti-PD1 (200 μg) and anti-CTLA4 (100 μg) were given intraperitoneally. Primary tumors were injected with NBTXR3 on day 6 and irradiated with 12-Gy (HDXRT) on days 7, 8, and 9; secondary tumors were irradiated with 1-Gy (LDXRT) on days 12 and 13. The survivor mice at day 178 were rechallenged with 344SQR cells and tumor growth monitored thereafter.

**Results:**

NBTXR3  +  HDXRT  +  LDXRT  +  ICIs had significant antitumor effects against both primary and secondary tumors, improving the survival rate from 0 to 50%. Immune profiling of the secondary tumors revealed that NBTXR3  +  HDXRT  +  LDXRT increased CD8 T-cell infiltration and decreased the number of regulatory T (Treg) cells. Finally, none of the re-challenged mice developed tumors, and they had higher percentages of CD4 memory T cells and CD4 and CD8 T cells in both blood and spleen relative to untreated mice.

**Conclusions:**

NBTXR3 nanoparticle in combination with radioimmunotherapy significantly improves anti-PD1 resistant lung tumor control via promoting antitumor immune response.

**Graphical Abstract:**

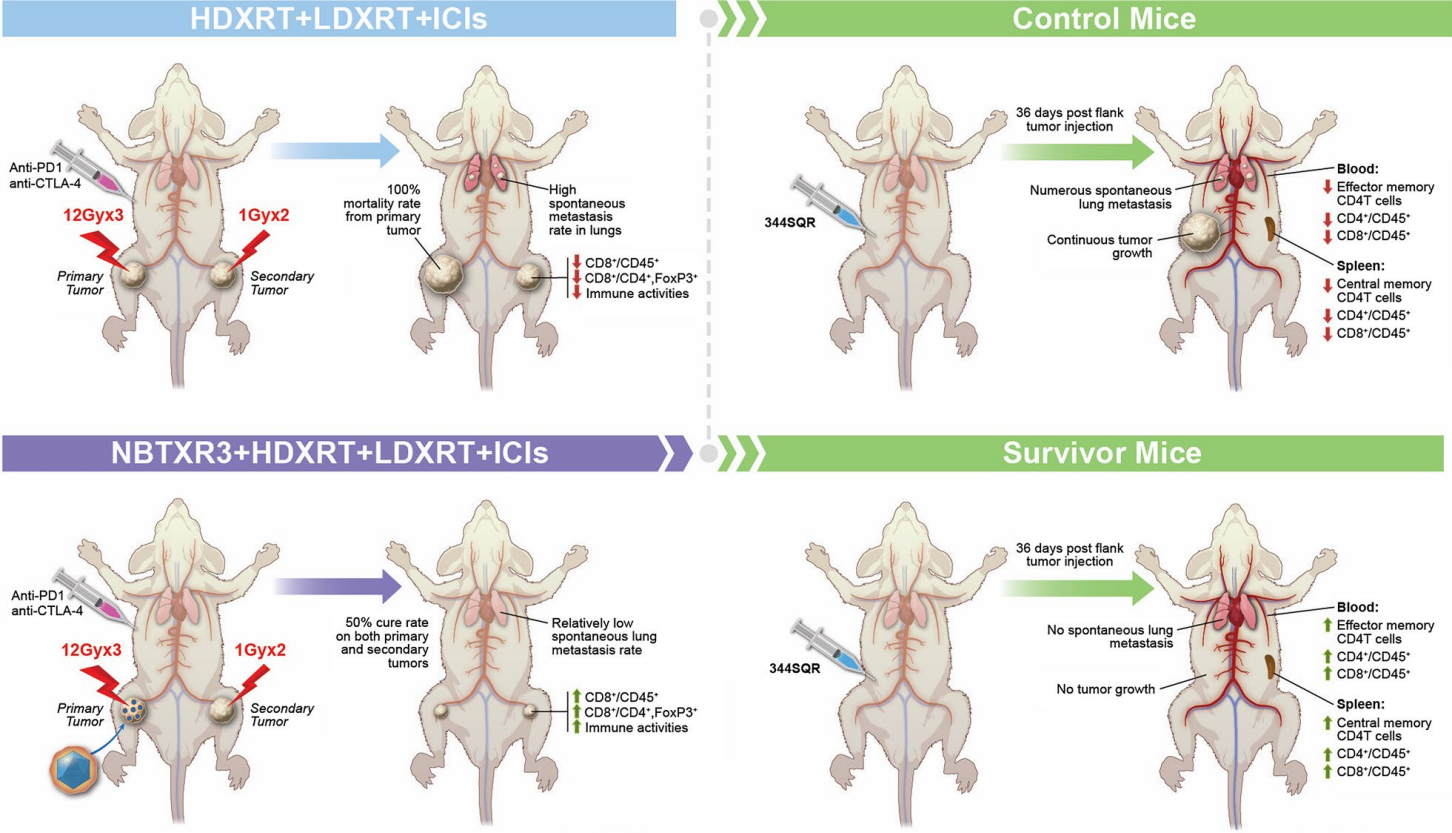

**Supplementary Information:**

The online version contains supplementary material available at 10.1186/s12951-021-01163-1.

## Introduction

Lung cancer accounts for nearly 25% of all cancer deaths worldwide [1], and the 5-year survival rates for metastasized lung cancer is only 5% [2]. In many cases, lung cancer cells have already metastasized at the time of diagnosis, and most patients die of metastatic disease [3]. Therefore, it is critical to develop therapies for effectively treating metastatic lung cancer. Radiation therapy has traditionally been considered a means of local tumor control through its direct effects on cancer cells; however, radiation also has systemic antitumor effects thought to be mediated by the immune system [4–6]. We previously found that delivering high radiation doses to primary tumors in combination with immune checkpoint blockade in a mouse model of metastatic lung cancer could induce potent systemic antitumor immune responses; we further showed that the addition of low doses of radiation to secondary tumors could modulate the immune microenvironment of those tumors [7]. However, this high and low dose radiation strategy was only evaluated in an anti-PD1 sensitive mouse model. It would be of great significance to validate it in an anti-PD1 resistant model, since most of the cancer patients are insensitive to anti-PD1 treatment. In addition, reasoning that augmenting and optimizing these local and systemic immune effects would enhance the effectiveness of this strategy, we explored the effects of adding NBTXR3, a hafnium oxide radioenhancing nanoparticle, to this dual-radiation-dose plus checkpoint blockade therapy in a mouse model of anti-PD1 resistant metastatic lung cancer. NBTXR3, which has been approved for the treatment of localized sarcoma [8], increases radiation-mediated damage to cancer cells and promotes activation of the STING pathway [9, 10], both of which could contribute to immune priming. We further found that the addition of NBTXR3 to radiation-plus-checkpoint blockade therapy also promoted abscopal effects in the metastatic lung cancer model [11]. However, that combination of localized radiation, NBTXR3, and immunotherapy did not completely eradicate the tumors. Given the potential immune-enhancing capacity of NBTXR3, we hypothesized that integrating these nanoparticles with a high-dose-plus-low-dose radiation strategy (Radscopal™), with dual immune checkpoint blockade, would enhance survival, promote systemic antitumor immune responses, and eliminate metastases in this mouse model.

## Materials and methods

### Materials

The NBTXR3 nanoparticles were provided by Nanobiotix and were kept at room temperature in a dark environment. The mouse anti-PD1 antibody (αPD1) (mPD1-4H2-mg1-D265aA) was kindly provided by Bristol-Myers Squibb. The mouse anti-CTLA4 antibody (αCTLA4) (cat. #BP0164) was purchased from BioXCell.

### Cell line and culture conditions

The anti-PD1-resistant lung cancer cell line 344SQR, created as described elsewhere [12], was used for all experiments. The 344SQR cells were cultured in RPMI-1640 medium supplemented with 10% fetal bovine serum and penicillin/streptomycin and incubated at 37 °C in a 5% CO_2_ atmosphere.

### Tumor inoculation and treatment

**Fig. 1:**
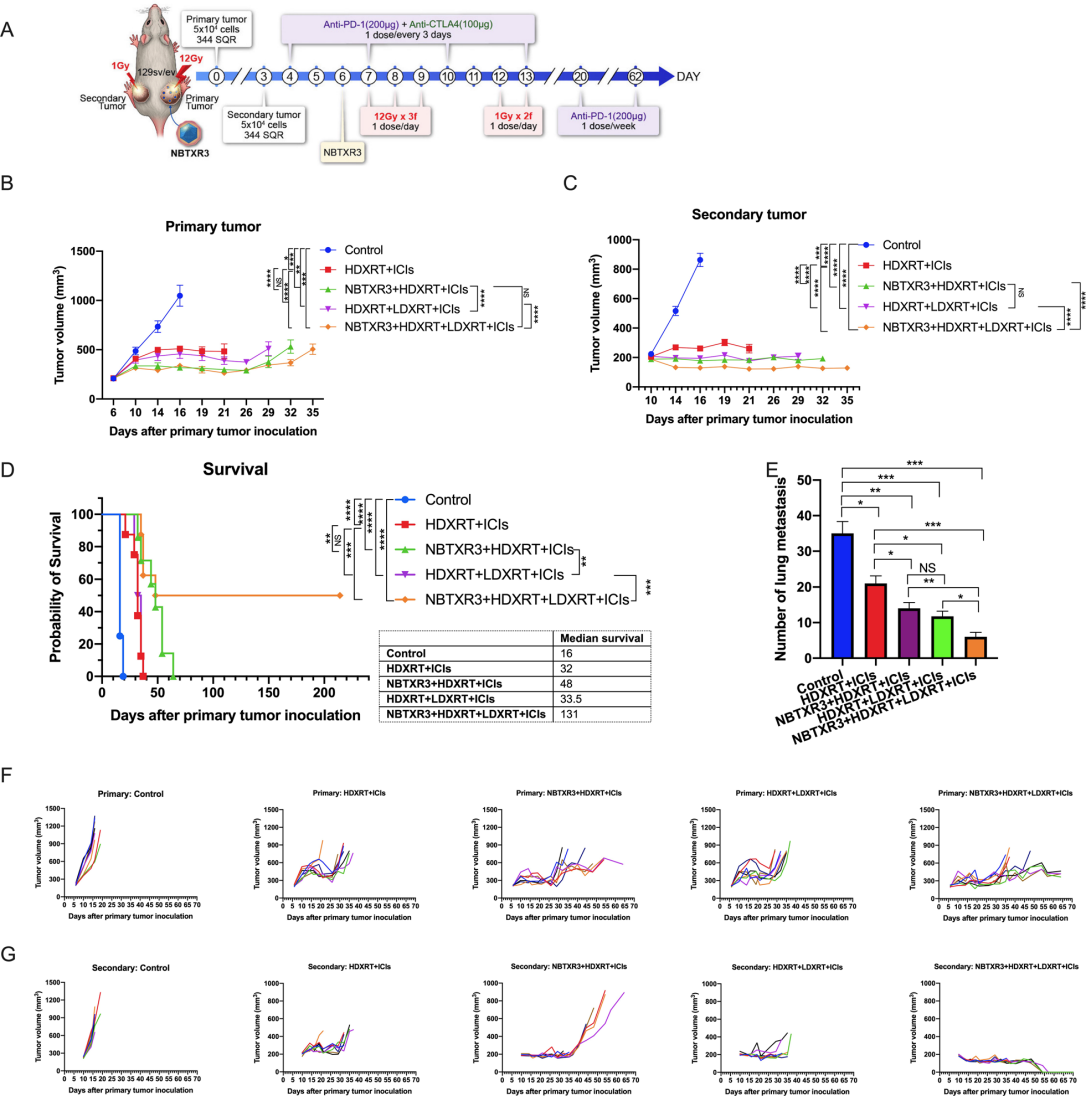
Treatment outcomes after therapy with NBTXR3, high- and low-dose radiotherapy, and immunotherapy. **A **Treatment schema for NBTXR3 given with high-dose and low-dose radiotherapy. Mice were subcutaneously inoculated with 5 × 10^4^ 344SQR cells in the right legs on day 0 (to establish primary tumors) and in the left legs on day 3 (to establish secondary tumors). NBTXR3 was delivered to the primary tumor by intratumoral injection on day 6. Primary tumors were treated with three 12-Gy fractions on day 7, 8, and 9 (HDXRT). Secondary tumors were irradiated with two 1-Gy fractions on day 12 and 13 (LDXRT). Anti-PD1 (200 μg) and anti-CTLA4 (100 μg) were given by intraperitoneal injection on days 4, 7, 10, and 13, and anti-PD1 treatment was continued once a week from day 20 until day 62. **B** Changes in primary tumor volumes over time. **C** Changes in secondary tumor volumes over time. Data are shown as means for each treatment group (n  = 7 or 8), with bars indicating standard error of the mean. **D** Survival rates and median survival times for each treatment group. **E** Number of spontaneous lung metastases on day 16 in each treatment group. All of the mice were injected with both anti-PD1 and anti-CTLA4, and all mice were euthanized when the any tumor exceeded 14 mm in diameter. **F** Individual primary tumor volumes for each treatment group. **G** Individual secondary tumor volumes for each treatment group. Data are expressed as means  ±  standard error of the mean (SEM). *P * < 0.05 was considered statistically significant. *P  < 0.05, **P  < 0.01, ***P  < 0.001, ****P  < 0.0001, *NS* not significant. *HDXRT* high-dose radiotherapy; *LDXRT* low-dose radiotherapy; *ICIs* immune checkpoint inhibitors

Mice in this study were 8- to 12-week-old 129/SvEv syngeneic females, purchased from Taconic Biosciences. The 344SQR cells [5 × 10^4^ in 100 μL phosphate-buffered saline (PBS)] were subcutaneously injected into the right legs of the mice on day 0 [to create the “primary” tumor, to be treated with high-dose radiation (HDXRT)] and into the left legs on day 3 [to form the “secondary” tumor, to be treated with low-dose radiation (LDXRT)]. Tumors were measured with digital calipers at least twice a week starting from day 6, and the tumor volumes were calculated as V  =  0.5 × width^2^ ×  length. All mice were given intraperitoneal injections of immune checkpoint inhibitors (ICIs), including anti-PD1 (200 μg/mouse) plus anti-CTLA4 (100 μg/mouse) on days 4, 7, 10, and 13, and the anti-PD1 (200 μg/mouse) was continued once a week from day 20 until day 65. The treatment schedule is shown in Fig. 1A. The five treatment groups were as follows: untreated (control), HDXRT  +  ICIs, HDXRT  +  LDXRT  +  ICIs, NBTXR3  +  HDXRT  +  ICIs, and NBTXR3  +  HDXRT  +  LDXRT  +  ICIs, with treatment conditions defined as follows. As described in a previous study [11], primary tumors of the HDXRT groups were treated with 3 fractions of 12 Gy each, delivered with an X-RAD 225Cx small-animal irradiator on days 7, 8, and 9 (total dose 36 Gy). The secondary tumors of the LDXRT groups were treated with 2 fractions of 1 Gy each, also delivered with an X-RAD 225Cx small-animal irradiator on day 12 and 13 (total dose 2 Gy). The dose was delivered with 2 opposing beams from anteroposterior and posteroanterior positions and a 15-mm circular collimator. Dosimetric measurements and treatment planning were done with a MATLAB-based system developed by our in-house radiation physics team at MD Anderson Cancer Center. All collimators were commissioned by Precision XRay Corporation at the time of installation, and routine output checks were done with an ion chamber to ensure that the outputs had not changed and that the treatment plans continued to be accurate. The NBTXR3 groups received intratumoral injection of NBTXR3 (60.8 mg/mL) in 5% glucose to 25% of the tumor volume on day 6. Mice were euthanized when any tumor (primary or secondary) reached 14 mm in any dimension. All animal procedures followed the guidelines of the Institutional Animal Care and Use Committee at MD Anderson Cancer Center.

### Tumor rechallenge

Mice from the NBTXR3  +  HDXRT  +  LDXRT  +  ICIs group that were still alive at day 178, and four untreated, 34- to 38-week-old control mice, were given subcutaneous injections of 5 × 10^4^ 344SQR cells in 100 μL PBS in the right flank. No further treatment was given. Tumor growth was monitored until the tumors reached 14 mm in longest diameter. All mice were euthanized 36 days after tumor inoculation, and lungs, spleen, and blood samples were harvested to count the numbers of lung metastases and to obtain profiles of CD4 and CD8 memory T cells.

### Tumor processing

Secondary tumors were harvested on day 19 for NanoString analyses, and on day 16 for flow cytometry analysis. Tumor tissues were cut into small pieces and digested with 250 µg/mL of Liberase (Roche, cat. #05401127001) and 20 µg/mL DNAse (Sigma-Aldrich, cat. #4716728001) at 37 °C for 30 min. The digestion process was stopped with 1 mL fetal bovine serum and the samples were filtered. Tumor-infiltrating lymphocytes (TILs) were enriched by using Histopaque 1077 (cat. #10771, Sigma-Aldrich). RNA was extracted by using a RNeasy Mini Kit (QIAGEN, cat. #74106) according to the manufacturer’s protocol, and the RNA was used for NanoString analysis as described below.

### NanoString analysis of immune-related genes

At least 50 ng of the RNA extracted from each secondary tumor was analyzed with an nCounter PanCancer Immune Profiling Panel and an nCounter MAX Analysis System (both from NanoString Technologies, Seattle, WA, USA) according to the manufacturer’s instructions; data were processed with the PanCancer Immune Profiling Advanced Analysis Module (also from NanoString Technologies). Higher immune scores indicate a greater abundance of immune cells.

### Flow cytometry analysis

For these analyses, secondary tumors were obtained on day 16, weighed, digested with Librase and DNAse, and the numbers of cells were counted with a TC20 Automated Cell Counter (Bio-Rad). Cells were then stained with anti-CD45-APC (cat. #103112), anti-CD3-BV510 (cat. #100234), anti-CD4-PE (cat. #100408), and anti-CD8-PercpCy5.5 (cat. #100734) (all from BioLegend). For intracellular staining of the Treg marker FOXP3, cells were fixed and permeabilized according to the manufacturer’s instructions (BioLegend) and stained with anti-Foxp3-Alexa 488 (cat. #126406).

Splenocytes and blood samples from the tumor re-challenge experiment were stained with anti-CD45-Pacific Blue (cat. #103126), anti-CD4-APC-Fire 750 (cat. #100460), anti-CD8-PercpCy5.5 (cat. #100734), anti-CD62L-PE-Cy7 (cat. #104418), and anti-CD44-APC (cat. #103012) (all from BioLegend). Samples were analyzed with a Gallios Flow Cytometer (Beckman Coulter) with Kaluza software Version 2.1.

### Analysis of numbers of lung metastases

Lungs were harvested either on day 16, or, in the tumor rechallenge experiment, from both the control group and the NBTXR3  +  HDXRT  +  LDXRT  +  ICIs group on day 36 after the rechallenge. The extracted lungs were stored in Bouin’s fixative solution (Polysciences Inc., cat. #16045-1) for 3 days, after which lung metastatic nodules were counted.

### TCR repertoire analysis

Four groups of 4 mice were treated with HDXRT  +  ICIs, HDXRT  +  LDXRT  +  ICIs, NBTXR3  +  HDXRT  +  ICIs, and NBTXR3  +  HDXRT  +  LDXRT  +  ICIs as described above. Total RNA was extracted from secondary tumors on day 19. TCR sequencing was performed by a method described in a previous study [11].

## Statistical analyses

All statistical analyses were done with Prism 8.0 (GraphPad Software). Tumor growth curves were compared by two-way analysis of variance and were expressed as mean tumor volume  ±  standard error of the mean (SEM). Mouse survival rates were analyzed with the Kaplan–Meier method, and estimates were compared with log-rank tests. The Nanostring data of Total TILs score and CD8 T cells score were analyzed with one-way ANOVA, and the Treg score and dendritic cell score were analyzed with Kruskal–Wallis test. All other data were analyzed with two-tailed t tests and expressed as mean value  ±  SEM. P values of  <  0.05 were considered to indicate statistically significant differences.

## Results

### NBTXR3 nanoparticle in combination with high and low-dose radiation improves tumor control and reduces the number of spontaneous lung metastases

Mice that received radiation had both primary and secondary tumors that were both smaller and grew more slowly than in the control mice (Fig. 1B). Further, consistent with our previous findings [11], primary tumors treated with both NBTXR3 and radiation grew more slowly than those treated with radiation alone (*P*  < 0.0001; Fig. 1B). Moreover, HDXRT to the primary tumor delayed the growth of the secondary tumors relative to the control (*P*  < 0.0001; Fig. 1C), thereby indicating an abscopal effect; giving NBTXR3 with HDXRT  +  ICIs further slowed growth of the secondary tumor relative to HDXRT  +  ICIs alone (*P * < 0.0001; Fig. 1C). The addition of LDXRT also delayed secondary tumor growth (HDXRT  +  LDXRT  +  ICIs vs. HDXRT  +  ICIs, *P * < 0.0001; NBXR3  +  HDXRT  +  LDXRT  +  ICIs vs. NBTXR3  +  HDXRT  +  ICIs, *P*  < 0.0001; Fig. 1C). As shown in Additional file 1: Fig. S1, although NBTXR3  +  HDXRT  +  LDXRT considerably reduced the growth of the primary tumor, it had little effect over the secondary tumor. Notably, the combination therapy (NBTXR3  +  HDXRT  +  LDXRT  +  ICIs) completely eradicated both the primary and secondary tumors in 4 of the 8 of the mice in that treatment group. The results indicate that ICIs are essential for controlling the secondary tumors.

The addition of NBTXR3 was also beneficial in terms of extending median survival times, from 16 days for the control group to 32 days for HDXRT  +  ICIs only, 33.5 days for HDXRT  +  LDXRT  +  ICIs, 48 days for NBTXR3  +  HDXRT  +  ICIs, and 131 days for the NBTXR3  +  HDXRT  +  LDXRT  +  ICIs group (Fig. 1D). In terms of tumor-related survival, NBTXR3 may also have improved rates of death from primary and secondary tumors: all of the mice in the control group (n  = 8), the HDXRT  +  ICIs group (n  = 8), and the HDXRT  +  LDXRT  +  ICIs group (n  = 8) were expired due to the growth of the primary tumor, i.e., the primary tumor exceeded 14 mm in greatest diameter; but only 3 of 7 mice in the NBTXR3  +  HDXRT  +  ICIs group and 4 of 8 mice in the NBTXR3  +  HDXRT  +  LDXRT  +  ICIs groups died from the primary tumor (Fig. 1F). In terms of death from the secondary tumors, 4 of the 7 mice in the NBTXR3  +  HDXRT  +  ICIs group died, but none of the 8 mice in the NBTXR3  +  HDXRT  +  LDXRT  +  ICIs group expired because of growth of the secondary tumor (Fig. 1G). Finally, the combination therapy also led to fewer spontaneous lung metastases: the NBTXR3  +  HDXRT  +  LDXRT  +  ICIs group had 6 ± 2 lung metastases as compared with 12 ± 2 for the HDXRT  +  LDXRT  +  ICIs group, 14 ± 1 for the NBTR3  +  HDXRT  +  ICIs group, 21 ± 3 for the HDXRT  +  ICIs group, and 35 ± 4 for the control group (Fig. 1E). These results suggest that the combination therapy was also effective in reducing the number of spontaneous lung metastases in this model.

### NBTXR3  +  HDXRT  +  LDXRT  +  ICIs modulates expression of immune-related antitumor genes

**Fig. 2:**
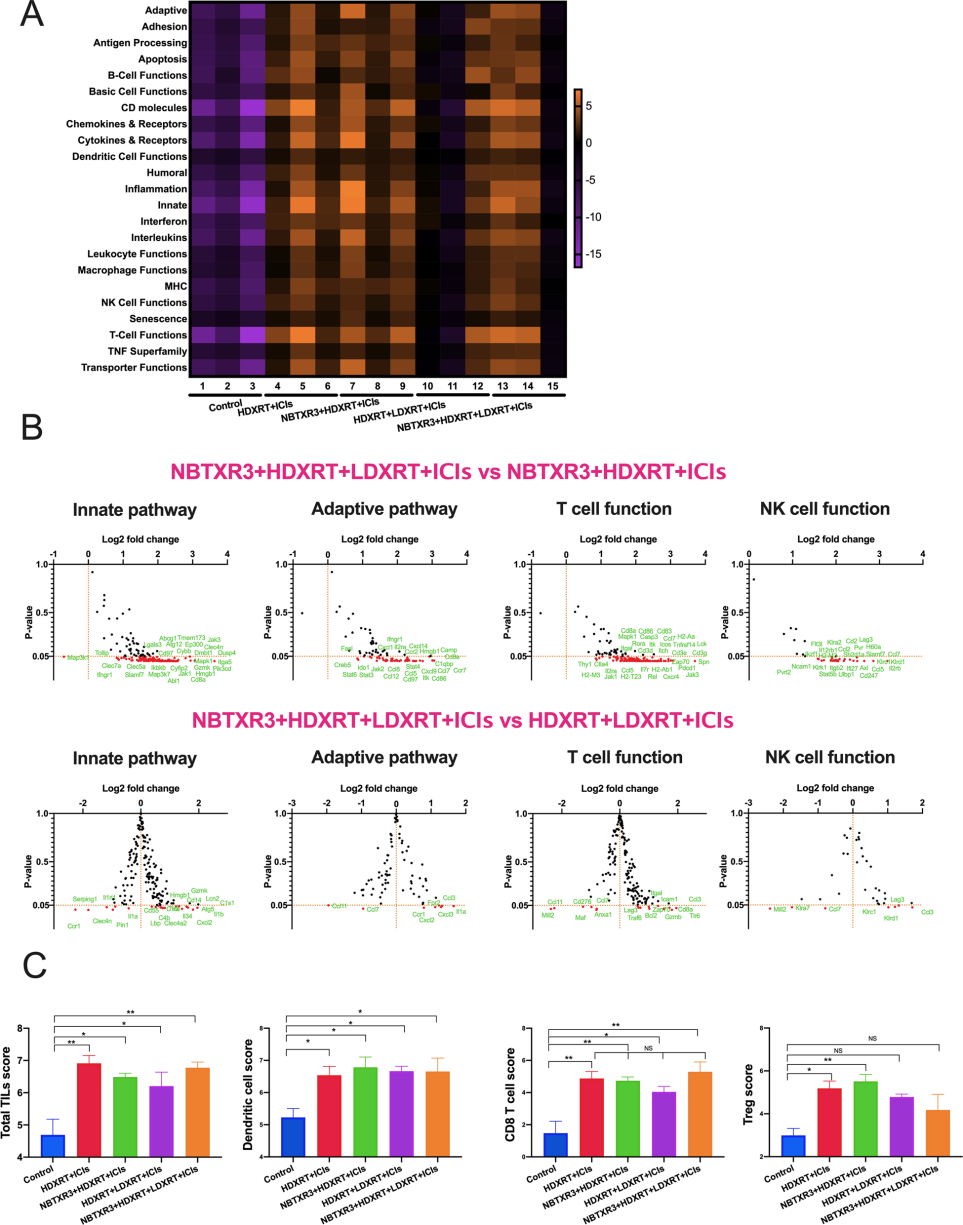
NanoString analysis of immune-gene expression in the secondary tumors. **A** Heatmap of the activities of various immune pathways. **B** Changes in gene expression in innate pathway, adaptive pathway, T cell and NK cell function in the NBTXR3  +  HDXRT  +  LDXRT  +  ICIs group relative to the NBTXR3  +  HDXRT  +  ICIs group and the HDXRT  +  LDXRT + ICIs group. **C** Relative scores for total tumor-infiltrating lymphocytes (TILs), dendritic cells, CD8 T cells, and regulatory T (Treg) cells. Data are expressed as means  ±  standard error of the mean (SEM). *P*  < 0.05 was considered statistically significant. *P  < 0.05, **P  < 0.01, ***P  < 0.001, *NS* not significant. *HDXRT* high-dose radiotherapy; *LDXRT* low-dose radiotherapy; *ICIs* immune checkpoint inhibitors

Next, we investigated how NBTXR3 and LDXRT affect immune-related gene expression patterns (and, theoretically, control of the secondary tumors). NanoString analysis of the RNA in immune cells from the secondary tumors (extracted at day 19) showed that all of the treatment conditions (relative to the control conditions) upregulated the activity of immune pathways involved in adaptive immune response, innate immune response, antigen processing, T-cell function, NK-cell function, and dendritic cell function (Fig. 2A). NBTXR3  +  HDXRT  +  LDXRT  +  ICIs seemed to have increased the activity of these immune pathways to a greater extent than did HDXRT  +  LDXRT  +  ICIs (Fig. 2A), which is consistent with our previous finding that NBTXR3 could upregulate major immune pathways [11]. However, LDXRT to the secondary tumors in this study seemed to suppress the activities of these immune pathways. The comparison of individual gene expression (Fig. 2B) reveals that the low dose radiation on the secondary tumor significantly upregulated expression of the genes, such as Cd8a, Ifngr1, GzmK, Cd86, Cd83, Casp3, etc. The addition of NBTXR3 to high- and low-dose radiation clearly affected expression of genes, such as Gzmb, Cd8a, Itgal, Ccl3, Il1a, etc., which are associated with T-cell and NK-cell function, innate immunity, and adaptive immunity (Fig. 2B). Remarkably, the addition of NBTXR3 to HDXRT  +  LDXRT  +  ICIs downregulated Atg5 in the secondary tumors. It has been reported that the knockout of ATG5 polarized macrophages to M1 phenotype, which increased inflammatory response [13]. In addition, NBTXR3 also upregulated Il1a expression. IL-1α, which can be expressed by macrophages, has been shown to strongly induce apoptosis [14, 15]. IL-1 α mediated-anti-tumor immune response may also develop into immunological memory against cancer cells [16]. In terms of numbers of each immune cell type, all of the treatments led to increases in TIL score, dendritic cell score, and CD8 T-cell score relative to the control condition (Fig. 2C). As for numbers of Tregs, the HDXRT  +  ICIs and NBTXR3  +  HDXRT  +  ICIs groups had more Tregs in the secondary tumor than the control group. In contrast, the numbers of Tregs in the HDXRT  +  LDXRT  +  ICIs and NBTXR3  +  HDXRT  +  LDXRT  +  ICIs groups were similar to that in the control group.

### Adding NBTXR3 nanoparticle to high- and low-dose radiation shifted immune cell subpopulations to favor tumor control

**Fig. 3:**
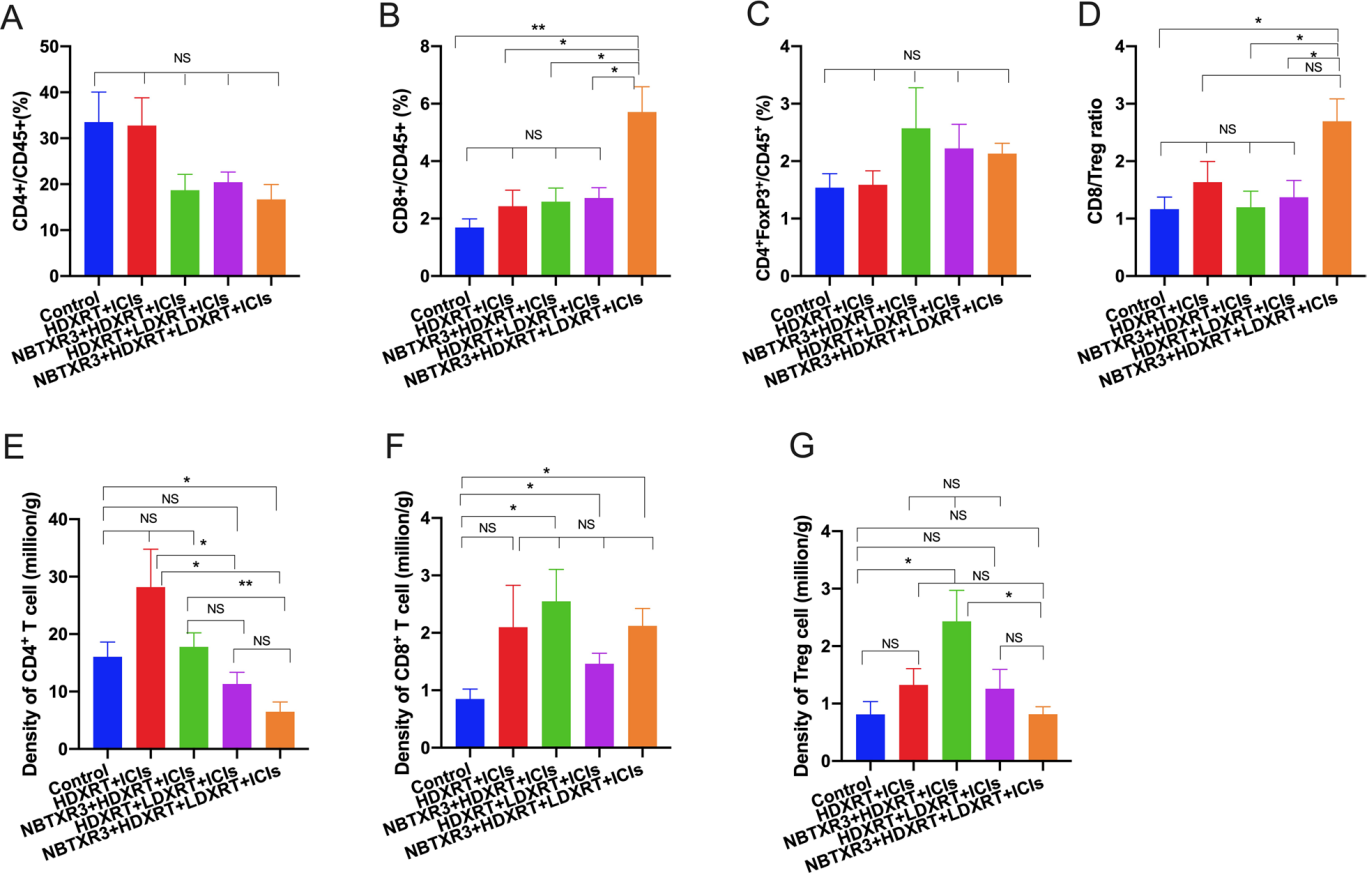
Flow cytometry analysis of CD4^+^ T cells, CD8^+^ T cells, and regulatory T cells (Tregs, labeled with CD4^+^FoxP3^+^) in the secondary tumors, harvested on day 16. **A** CD4^+^ T cell/CD45^+^ cell ratio. **B** CD8^+^ T cell/CD45^+^ cell ratio. **C** Treg CD4^+^FoxP3^+^ cell/CD45^+^ cell ratio. **D** CD8^+^ T cell/Treg CD4^+^FoxP3^+^ cell ratio. **E** Density of CD4^+^ T cells. **F** Density of CD8^+^ T cells. **G** Density of Treg CD4^+^FoxP3^+^ cells. Data are expressed as means  ±  standard error of the mean (SEM). *P*  < 0.05 was considered statistically significant. *P  < 0.05, **P  < 0.01, ***P  < 0.001, ****P  < 0.0001, *NS* not significant. *HDXRT* high-dose radiotherapy; *LDXRT* low-dose radiotherapy; *ICIs* immune checkpoint inhibitors

We further confirmed the shift in proportions of the various subtypes of immune cells from the secondary tumors via flow cytometry analysis. None of the various treatments significantly affected the CD4^+^/CD45^+^ ratio (Fig. 3A) or the Treg CD4^+^FoxP3^+^ /CD45^+^ ratio (Fig. 3C). However, NBTXR3  +  HDXRT  +  LDXRT  +  ICIs increased the percentage of CD8^+^ T cells (to 5.71 ± 0.88%) over that in the other groups (Fig. 3B). Also, in the secondary tumors, the NBTXR3  +  HDXRT  +  LDXRT  +  ICIs treatment led to a significantly higher CD8 T cell/Treg ratio (2.70 ± 0.39) than the control (1.16 ± 0.21), HDXRT  +  LDXRT  +  ICIs (1.37 ± 0.29), and NBTXR3  +  HDXRT  +  ICIs (1.20 ± 0.28) conditions (Fig. 3D). Unexpectedly, the HDXRT  +  LDXRT  +  ICIs and NBTXR3  +  HDXRT  +  LDXRT  +  ICIs groups had less dense CD4 T cells than in the HDXRT  +  ICIs and NBTXR3  +  HDXRT  +  ICIs groups, respectively (Fig. 3E), suggesting that LDXRT negatively affects the infiltration of CD4 T cells into the secondary tumors. With regard to CD8^+^ T cell density, the NBTXR3  +  HDXRT  +  ICIs condition led to the highest density (2.55 ± 0.56 million/g), followed by the HDXRT  +  LDXRT  +  ICIs (1.46 ± 0.18 million/g) and NBTXR3  +  HDXRT  +  LDXRT  +  ICIs (2.12 ± 0.31 million/g) condition, and all were higher than in the control condition (0.85 ± 0.17 million/g) (Fig. 3F). The NBTXR3  +  HDXRT  +  ICIs condition also increased the density of Tregs (2.43 ± 0.54 million/g) relative to the control (0.81 ± 0.23 million/g), but the NBTXR3  +  HDXRT  +  LDXRT  +  ICIs condition decreased the density of Tregs (0.82 ± 0.13 million/g) relative to the NBTXR3  +  HDXRT  +  ICIs group (Fig. 3G). These results suggest that the antitumor effectiveness of NBTXR3  +  HDXRT  +  LDXRT  +  ICIs could have resulted from enhanced infiltration of CD8  +  T cells and a favorable CD8/Treg ratio.

### NBTXR3 nanoparticle, given with immunoradiation, reshapes the TCRβ repertoire

**Fig. 4:**
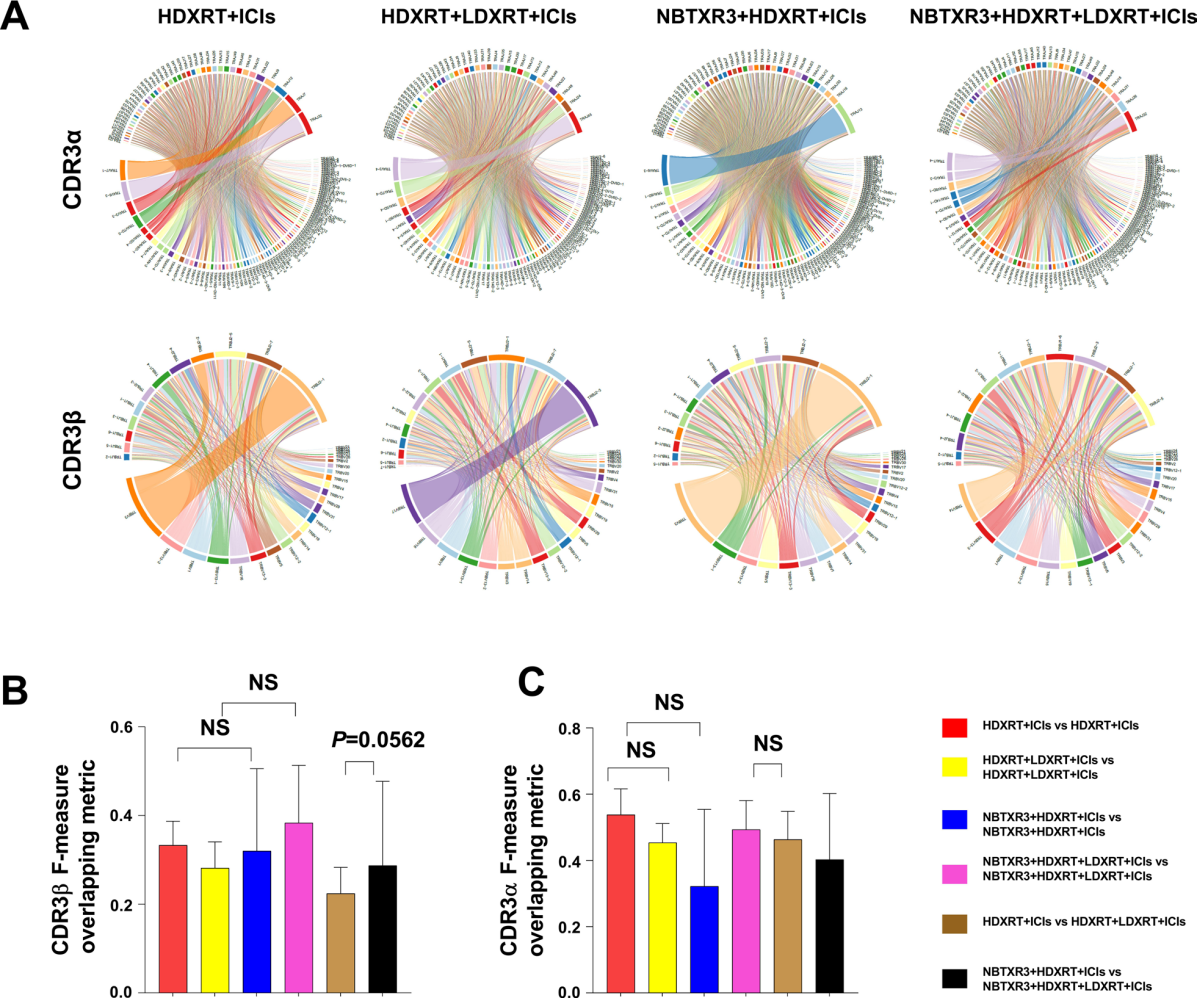
NBTXR3 increases the number of overlapping TCRβ receptors. **A** Representative Circos plots displaying the pairings of V-J gene families of the TCRβ and TCRα repertoires from all four treatment groups. Each stem connecting V and J gene pairs represent a unique CDR3 clonotype, and stem thickness is proportional to the CDR3 frequency. The lengths of the arcs are proportional to the gene family frequencies within each mouse repertoire. **B** Comparisons of overlapping, F-measure-normalized CDR3β frequencies within and between all four treatment groups. **C** Comparisons of overlapping, F-measure-normalized CDR3α frequencies within and between all four treatment groups Error bars represent means with standard deviation. *P*  < 0.05 from Mann–Whitney U tests was considered statistically significant. *P  < 0.05, **P  < 0.01, ***P  < 0.001, ****P  < 0.0001, *NS* not significant. *HDXRT* high-dose radiotherapy; *LDXRT* low-dose radiotherapy; *ICIs* immune checkpoint inhibitors

We analyzed the TCR repertoire of tumor-infiltrating T cells within the metastatic tumors for the effect of NBTXR3. Circos plots of TCR variable (V) and joining (J) gene family pairs of productive CDR3β and CDR3α are shown in Fig. 4A (representative figures from each group). To determine whether there was any difference in the diversity of the repertoires due to NBTXR3, we analyzed the normalized Shannon clonality indices between the NBTXR3 groups and their non-NBTXR3 counterparts but observed no differences in diversity for both CDR3β and CDR3α sequences (Additional file 2: Fig. S2), confirming our previous findings [11]. As a result, we hypothesized that the NBTXR3-induced tumor control that we observed in all NBTXR3-treated mice was likely mediated by tumor-specific T cells shared among the NBTXR3-treated mice. To test this hypothesis, we used the F measure, which is highly sensitive at grouping or differentiating TCR sequences based on degree of similarity [17], to compare the degree of overlapping sequences shared between the non-NBTXR3-treated groups with the clonotypes shared between the NBTXR3 groups. Among CDR3βs, we observed that NBTXR3 significantly increased the frequencies of shared clonotypes among the mice that received only high dose of radiation (NBTXR3  +  HDXRT  +  ICIs vs. HDXRT  +  ICIs, *P*  = 0.0476, Fig. 4B), but it did not significantly increase them among the mice treated with high and low dose (Radscopal™) radiation (NBTXR3  +  HDXRT  +  LDXRT  +  ICIs vs. HDXRT  +  LDXRT  +  ICIs, *P*  = 0.3095, Fig. 4B). Furthermore, NBTXR3 increased the number of overlapping CDR3β sequences shared between the “NBTXR3  +  HDXRT  +  ICIs” and “NBTXR3  +  HDXRT  +  LDXRT  +  ICIs” groups compared to those shared between these same groups in the absence of NBTXR3 (*P*  < 0.0001, Fig. 4B). None of these observations were made among the CDR3α however (Fig. 4C), which might be due to the presence of two TCRα per T cell [18]. These higher proportions of overlapping CDR3β sequences with NBTXR3 suggests that NBTXR3 might be inducing the expression of certain nanoparticle-associated epitopes that are recognized by the shared repertoires.

### Adding NBTXR3 nanoparticle to high- and low-dose radiation induces robust long-term immune memory

**Fig. 5:**
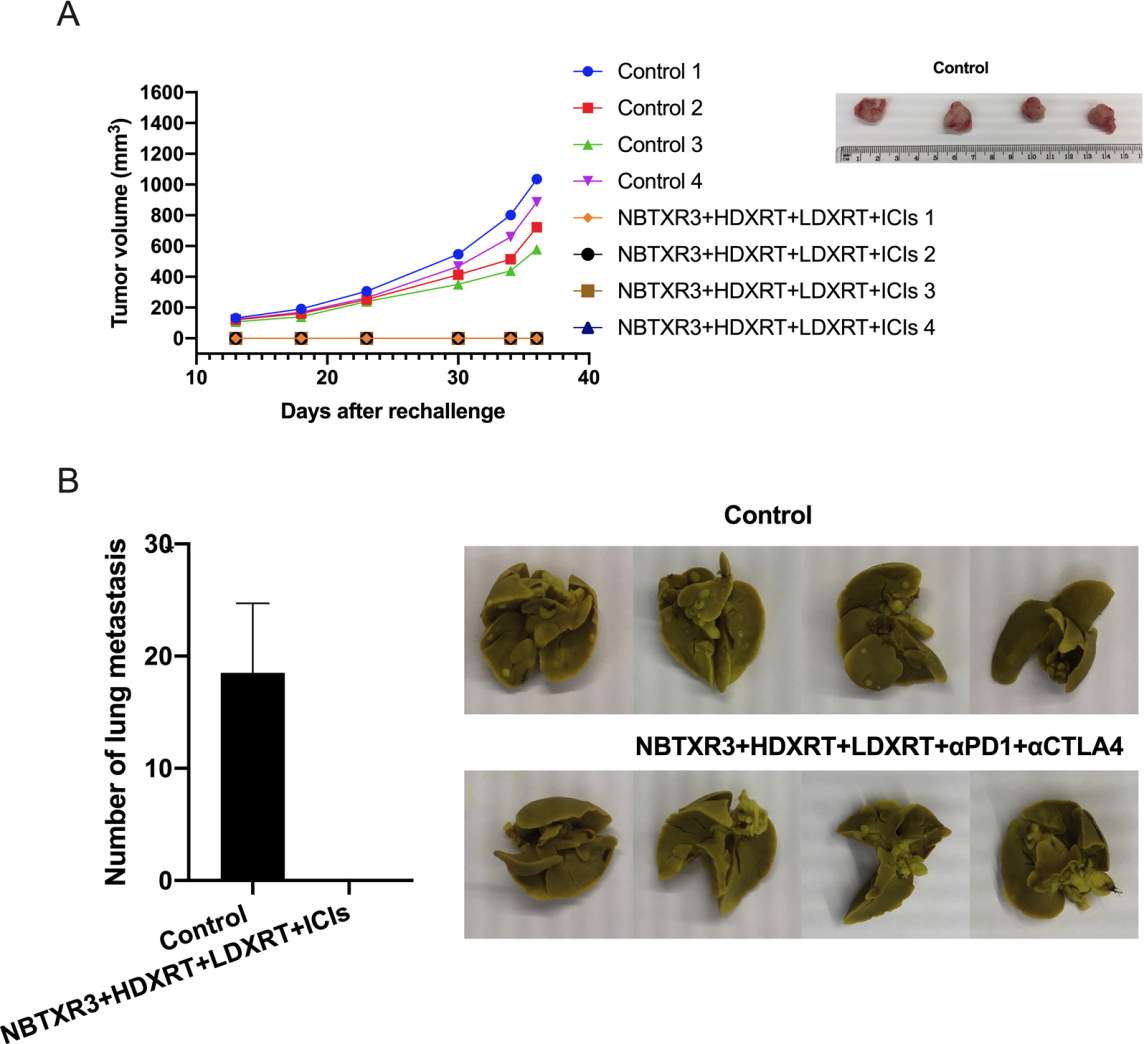
Tumor growth curves and lung metastases in mice rechallenged with 344SQR cells. **A** Left, tumor growth curves for each of the surviving mice from the NBTXR3  +  HDXRT  +  LDXRT  +  ICIs treatment group and the four control mice after rechallenge with 344SQR cells in the right flank. Right, images of excised tumors from the control group. **B** Numbers (left) and images (right) of lung metastases at 36 days after tumor rechallenge. Data are expressed as means  ±  standard error of the mean (SEM). *P*  <  0.05 was considered statistically significant. *P  <  0.05. *HDXRT* high-dose radiotherapy; *LDXRT* low-dose radiotherapy; *ICIs* immune checkpoint inhibitors

As noted earlier, 4 of the 8 mice in the NBTXR3  +  HDXRT  +  LDXRT  +  ICIs survived and showed no evidence of either primary or secondary tumors at the end of the first experiment, prompting the question of whether these mice had developed an effective immune memory against the lung cancer cells. To this end, the 4 mice from the NBTXR3  +  HDXRT  +  LDXRT  +  ICIs group that survived were re-challenged with 344SQR cells in the right flank at day 178. Another 4 untreated mice, aged 34–38 weeks (i.e., about the same age as the surviving mice) were also inoculated with 344SQR cells in the right flank to function as a control group. No treatment was given to any of these mice, and tumor growth was monitored for 36 days. Tumors in the control group grew steadily over that period (Fig. 5A), but no tumors were detected in the mice in the NBTXR3  +  HDXRT  +  LDXRT  +  ICIs group during that same period (Fig. 5A). On day 36 after the rechallenge, lungs were harvested from the mice and lung metastases counted. At that time, mice in the control group had a mean (±  SEM) of 19 ± 7 lung tumor nodules but the survivors from the NBTXR3  +  HDXRT  +  LDXRT  +  ICIs had no lung metastases (Fig. 5B). These results suggest that the surviving mice may have developed a systemic antitumor response.

**Fig. 6:**
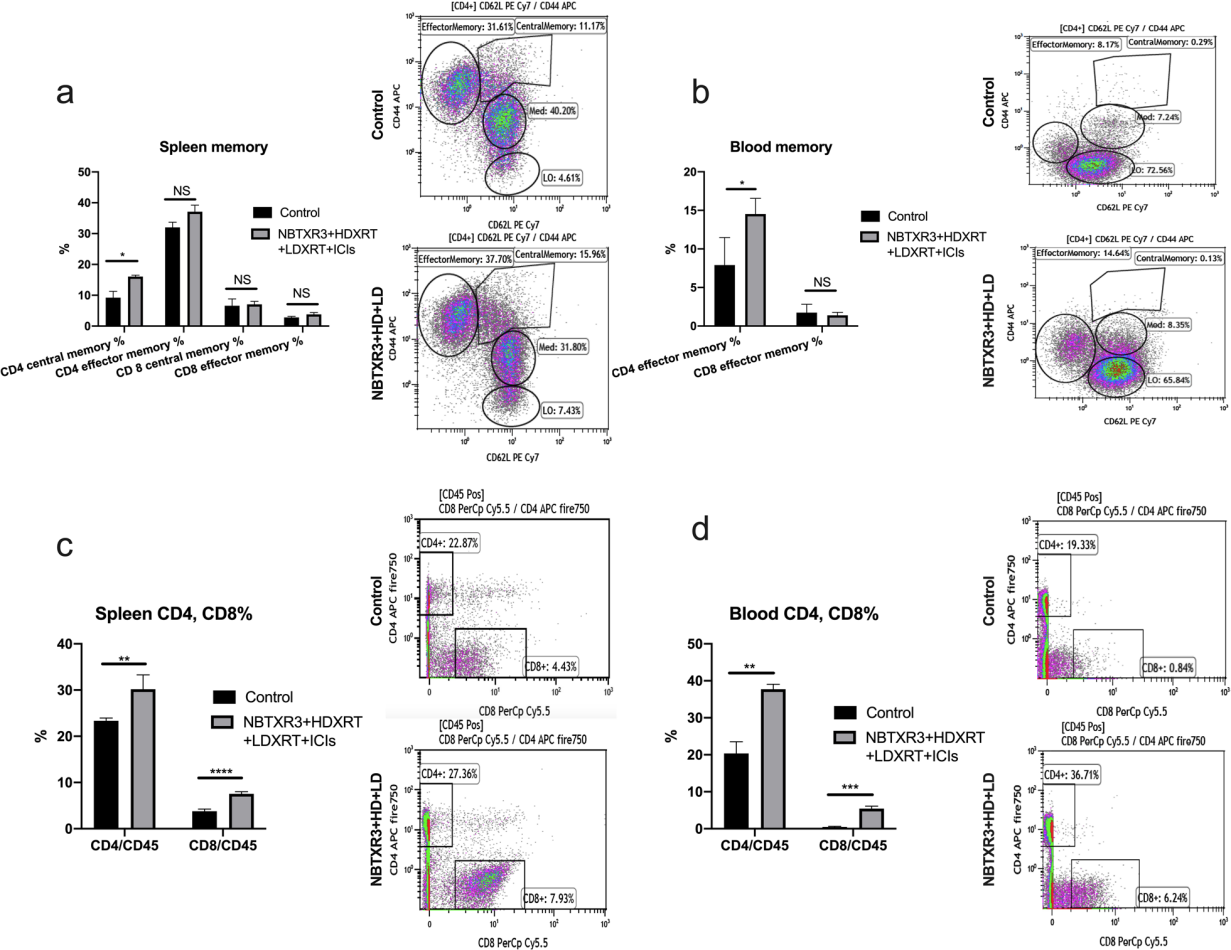
Flow cytometry analysis of memory CD8 T and CD4 T cells and total percentages of CD4 T and CD8 T cells in spleen and blood samples at 36 days after rechallenge. **A** Percentages of CD4 and CD8 memory T cells in spleen. **B** Percentages of CD4 and CD8 memory T cells in blood. **C** Percentages of CD4/CD45 and CD8/CD45 in spleen. **D** Percentages of CD4/CD45 and CD8/CD45 in blood. Data are expressed as means  ±  standard error of the mean (SEM). *P*  < 0.05 was considered statistically significant. *P  < 0.05, **P  < 0.01, ***P  < 0.001, ****P  < 0.0001, *NS* not significant. *HDXRT* high-dose radiotherapy; *LDXRT* low-dose radiotherapy; *ICIs* immune checkpoint inhibitors

Finally, to further explore the question of whether immune memory had been evoked in these mice after rechallenge, we used flow cytometry to analyze the proportions of CD4 and CD8 memory cells in spleen and blood samples from those mice at the end of the experiment. The NBTXR3  +  HDXRT  +  LDXRT group had more central memory CD4 T cells in the spleen than did the control group (16.1 ± 0.4% vs. 9.2 ± 2.0%) (*P*  < 0.05; Fig. 6A), and the NBTXR3  +  HDXRT  +  LDXRT  +  ICIs mice also had more effector memory CD4 T cells in the blood than did the controls (14.5 ± 1.0% vs. 7.9 ± 1.8%) (*P * < 0.05; Fig. 6B). Percentages of memory CD8 T cells in both spleen and blood were either too low to be measured or were no different between the control and NBTXR3  +  HDXRT  +  LDXRT  +  ICIs groups (Fig. 6A, B). In terms of percentages of CD4/CD45 and CD8/CD45 cells, in the spleen the NBTXR3  +  HDXRT  +  LDXRT mice had higher percentages of CD4 T cells (30.2 ± 1.6%) and CD8 T cells (7.6 ± 0.2%) than did the control group [CD4 T cells, 23.4 ± 0.3% (*P*  < 0.01); and CD8 T cells, 3.8 ± 0.2% (P  < 0.0001)] (Fig. 6C). In the blood, the NBTXR3  +  HDXRT  +  LDXRT mice also had significantly higher percentages of CD4 T cells (37.8 ± 1.3%) and CD8 T cells (5.4 ± 0.7%) than did the control mice [CD8 T cells, 0.5 ± 0.2% (*P*  < 0.01)]; and CD4 T cells [20.3 ± 3.2% (*P* < 0.001)] (Fig. 6D). The higher percentages of both CD4 and CD8 T cells in spleen and blood suggest that mice treated with NBTXR3  +  HDXRT  +  LDXRT  +  ICIs maintained a long-term adaptive immune response against tumor, which could be responsible for eradicating the re-injected cancer cells.

## Discussion

Outcomes after treatment for metastatic lung cancer depend mainly on effective control of the metastases [19, 20]. Combination radiation-plus-immune checkpoint inhibitors have emerged as a promising strategy for inducing a systemic antitumor immune response, a key component of effectively treating metastatic cancers [5]. We previously reported a radiotherapy schedule (RadScopal™) consisting of HDXRT to primary tumors and simultaneous LDXRT to secondary tumors that was developed to induce such an antitumor immune response with the goal of treating secondary (metastatic) tumors [7]. The HDXRT acts to prime the immune system at the primary tumor, and the LDXRT facilitates infiltration of anti-tumor lymphocytes and favors M1 macrophage polarization. In the current study, the integration of NBTXR3 into the high- and low-dose radiation strategy improved control of both the primary and secondary tumors as well as extending survival. Presumably the effects of NBTXR3 on the primary tumor result from its radiation-enhancing effect, as indicated in previous studies [21, 22]. The delay in growth of the secondary tumors and appearance of fewer lung metastases in the NBTXR3  +  HDXRT  +  LDXRT  +  ICIs mice suggest that this treatment induced a more potent systemic antitumor immune response than the HDXRT  +  LDXRT  +  ICIs strategy alone. We previously showed that giving NBTXR3 with XRT led to a significantly improved abscopal effect relative to XRT alone by increasing the number CD8 T cells and augmenting activities of various antitumor immune pathways [11]. In the current study, both NanoString and flow cytometry analyses of the secondary tumors confirmed that integrating NBTXR3 into dual-dose (HDXRT  +  LDXRT) radiation promoted the activities of major immune pathways, elevated the expression of a wide range of antitumor genes, increased the percentage of CD8 T cells, and increased the CD8 T cell/Treg ratio. CD8 T cells are known to have vital roles in antitumor immunity and thus having higher numbers of CD8 T cells would presumably enhance antitumor capacity [23]. Having higher CD8/Treg ratios has also been reported to correlate with better treatment outcomes [24, 25]. Collectively, these changes suggest that the addition of NBTXR3 created an immune environment that favors cancer killing. Moreover, because Tregs act as suppressor cells in terms of mounting an antitumor immune response [26, 27], the lower density of Tregs and higher CD8/Treg ratios in the NBTXR3  +  HDXRT  +  LDXRT  +  ICIs mice relative to the NBTXR3  +  HDXRT  +  ICIs mice suggest that LDXRT may reduce immune suppression by downregulating Tregs. This would be consistent with our previous finding that low-dose radiation (i.e., two 1-Gy fractions) could promote an antitumor immune response by reprogramming the immune microenvironment [7].

We studied the TCR repertoire of TILs from the metastatic sites to determine how differences in their amino acid sequences could explain the improved anti-tumoral effects that is observed with NBTXR3. We discovered that the two NBTXR3-treated mice subgroups shared more overlapping TCRβ sequences within each group compared to those shared within the corresponding non-NBTXR3 mice subgroups. These increases were significant only among the abscopally treated tumors (i.e., mice that received only high-dose radiation). This finding suggests that NBTXR3 might be inducing the expression of specific tumor epitopes that are recognized by primary site TILs with conserved TCRs that control metastatic disease after migration to those sites. The absence of significantly higher overlap frequencies with NBTXR3 among the high-and-low dose irradiated (Radscopal™) mice could be due to the generation of different TILs within the metastatic sites that are specific to epitopes induced by the low dose radiation. Overall, our TCR studies indicate that NBTXR3 reshapes the T cell repertoire and that might contribute to the improved antitumoral response with NBTXR3.

Relapses of lung cancer are common [28, 29], and thus the ability to evoke robust, long-term immune memory against recurring cancer cells would be considerably useful. The prevention of tumor growth in the surviving mice in the NBTXR3  +  HDXRT  +  LDXRT  +  ICIs group after tumor rechallenge illustrates that these mice may have acquired an effective and systemic antitumor immune memory status. Indeed, our flow cytometry findings confirmed that NBTXR3  +  HDXRT  +  LDXRT  +  ICIs led to significantly higher percentages of effector memory CD4 T cells in the blood and central memory CD4 T cells in the spleen. Most likely, the memory CD4 T cells initiated the memory immune response against the re-challenged tumors by facilitating activation and proliferation of CD8 T cells [30]. This hypothesis is supported by our discovery of higher percentages of CD8 T cells in both blood and spleen of the surviving mice. Logically, one might conclude that the memory CD4 T cells are responsible for effectively activating naïve CD8 T cells in response to the tumor rechallenge.

## Conclusion

In summary, we found that integrating NBTXR3 nanoparticle into a high-dose-plus-low-dose radiation strategy slowed the growth of both primary and secondary tumors, suppressed the appearance of lung metastases, and increased survival rates. NBTXR3 also enhanced the activities of major antitumor immune signaling pathways and increased the CD8/Treg ratio in the secondary tumors. Moreover, mice treated with NBTXR3  +  HDXRT  +  LDXRT  +  ICIs maintained potent, long-term antitumor immune memory that suppressed the growth of rechallenged tumors. This combination of NBTXR3, high-dose plus low-dose radiation, and immune checkpoint blockade may well improve treatment outcomes in metastatic lung cancer and reduce the side effects of systemic high-dose radiation in the clinic. We are currently in the process of testing whether this combination treatment strategy is effective in cancer patients at MD Anderson Cancer Center (Clinical trial.gov NCT03589339).

## Supplementary Information

**Additional file 1: Figure S1.** Treatment outcomes after therapy with NBTXR3, high- and low-dose radiotherapy. **A** Changes in primary tumor volumes over time. **B** Changes in secondary tumor volumes over time. Mice were subcutaneously inoculated with 5 x 10^4^ 344SQR cells in the right legs on day 0 (to establish primary tumors) and in the left legs on day 4 (to establish secondary tumors). NBTXR3 was delivered to the primary tumor by intratumoral injection on day 7. Primary tumors were treated with three 12-Gy fractions on day 8, 9, and 10 (HDXRT). Secondary tumors were irradiated with two 1-Gy fractions on day 13 and 14 (LDXRT).

**Additional file 2: Figure S2.** Normalized Shannon clonality comparison of CDR3β and CDR3α clonotypes between NBTXR3-treated mice and their corresponding non-NBTXR3 groups. P<0.05 from Mann-Whitney U tests was considered statistically significant. *NS* not significant.

## Data Availability

The data and materials that support the findings of this study are available from the corresponding author, upon reasonable request.
