# Supplementary figures and images for: A radioenhancing nanoparticle mediated immunoradiation improves survival and generates long-term antitumor immune memory in an anti-PD1-resistant murine lung cancer model
Source: J Nanobiotechnology. 2021 Dec 11;19:416. doi: 10.1186/s12951-021-01163-1 (PMC8666086; doi:10.1186/s12951-021-01163-1)

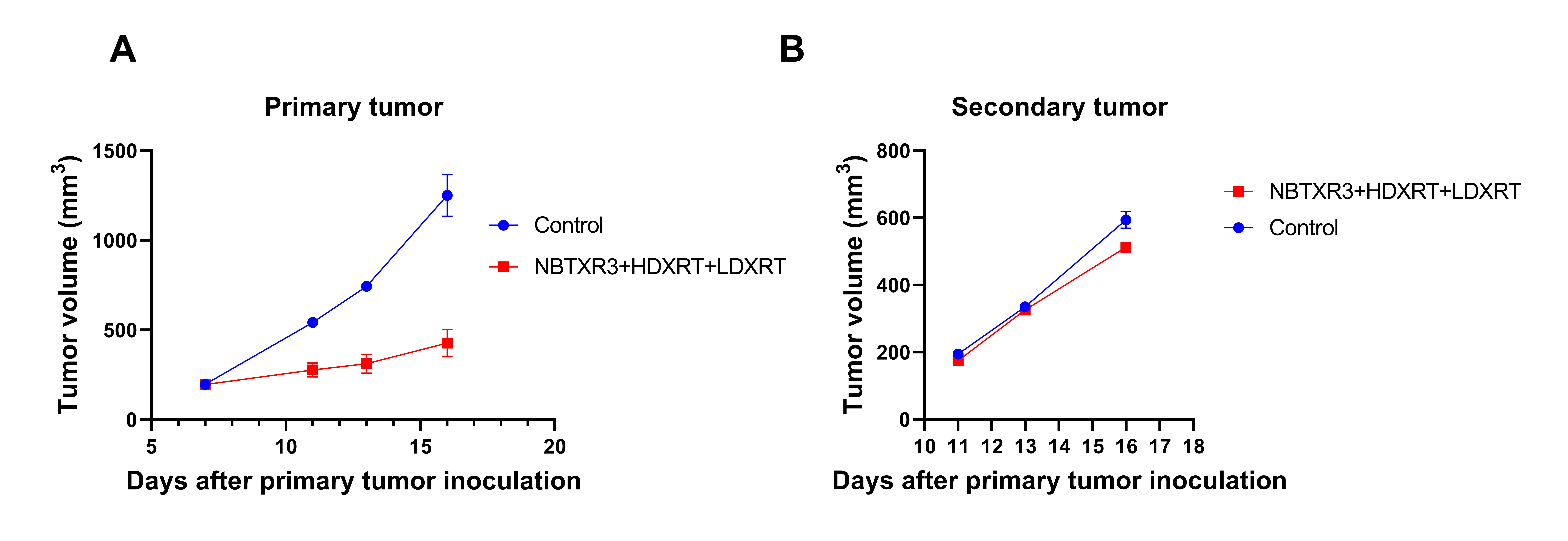

Supplement: Supplementary file 1 — Additional file 1: Figure S1. Treatment outcomes after therapy with NBTXR3, high- and low-dose radiotherapy. A Changes in primary tumor volumes over time. B Changes in secondary tumor volumes over time. Mice were subcutaneously inoculated with 5 x 10^4^ 344SQR cells in the right legs on day 0 (to establish primary tumors) and in the left legs on day 4 (to establish secondary tumors). NBTXR3 was delivered to the primary tumor by intratumoral injection on day 7. Primary tumors were treated with three 12-Gy fractions on day 8, 9, and 10 (HDXRT). Secondary tumors were irradiated with two 1-Gy fractions on day 13 and 14 (LDXRT). [file 12951_2021_1163_MOESM1_ESM.jpg]
